# Ononin Shows Anticancer Activity Against Laryngeal Cancer *via* the Inhibition of ERK/JNK/p38 Signaling Pathway

**DOI:** 10.3389/fonc.2022.939646

**Published:** 2022-07-14

**Authors:** Ben Ye, Jianhua Ma, Zhaoxia Li, Yang Li, Xiaopan Han

**Affiliations:** ^1^ Department of Ear, Nose, and Throat (ENT), Shandong Provincial Hospital Affiliated to Shandong First Medical University, Ji’nan, China; ^2^ Department of Cardiology, Shandong Rongjun General Hospital, Ji’nan, China; ^3^ Department of Plastic Surgery, Central Hospital Affiliated to Shandong First Medical University, Jinan, China; ^4^ Department of ENT, Central Hospital Affiliated to Shandong First Medical University, Ji’nan, China

**Keywords:** laryngeal cancer, ononin, Hep-2 cells, apoptosis, ERK/JNK/p38 pathway

## Abstract

**Background:**

Laryngeal cancer is a type of head and neck tumor with a poor prognosis and survival rate. The new cases of laryngeal cancer increased rapidly with a higher mortality rate around the world.

**Objective:**

The current research work was focused to unveil the *in vitro* antitumor effects of ononin against the laryngeal cancer Hep-2 cells.

**Methodology:**

The cytotoxic effects of ononin against the laryngeal cancer Hep-2 cells and normal HuLa-PC laryngeal cells were studied using an 3-(4,5-dimethylthiazol-2-yl)-2,5-diphenyl tetrazolium bromide (MTT) assay. The intracellular Reactive Oxygen Species (ROS) generation, apoptotic cell death, Mitochondrial Membrane Potential (MMP), and cell adhesion on the 25 and 50 µM ononin-treated Hep-2 cells were detected using respective staining assays. The levels of TBARS and antioxidants were assayed using specific kits. The expressions of c-Jun N-terminal kinase 1/2 (JNK1/2), Extracellular Signal-regulated Kinase 1/2 (ERK1/2), p38, Phosphatidylinositol-3 Kinase 1/2 (PI3K1/2), and protein kinase-B (Akt) in the ononin-treated Hep-2 cells were investigated using Reverse Transcription-Polymerase Chain Reaction (RT-PCR) assay.

**Results:**

The ononin treatment effectively inhibited the Hep-2 cell viability but did not affect the viability of HuLa-PC cells. Furthermore, the ononin treatment effectively improved the intracellular ROS accumulation, depleted the MMP, and triggered apoptosis in Hep-2 cells. The Thiobarbituric acid reactive substances (TBARS) were improved, and Glutathione (GSH) levels and Superoxide dismutase (SOD) were depleted in the ononin-administered Hep-2 cells. The ononin treatment substantially inhibited the JNK/ERK/p38 axis in the Hep-2 cells.

**Conclusion:**

Together, the outcomes of this exploration proved that the ononin has remarkable antitumor activity against laryngeal cancer Hep-2 cells.

## Introduction

Laryngeal cancer is a common head and neck tumor with increased occurrence and death rates. In 2018, approximately 177,000 new cases with 94,000 mortalities were recorded worldwide due to laryngeal cancer (1). The aged persons particularly men have a higher chance of developing laryngeal cancer (2). The cancer initiation is primarily triggered by the array of alterations in the cell genome. These alterations provoke the cells to incessantly multiplication and evade apoptosis, thus disturbing the tissue homeostasis (3).

Most of the signaling cascades that mediate the tumor cell apoptosis are Mitogen-Activated Protein Kinase (MAPK) family members (4). The Extracellular Signal-regulated Kinase (ERK), c-Jun N-terminal kinase (JNK), and p38 kinase are the well-known MAPK subfamily proteins. c-Jun N-terminal kinase (JNK) enhances apoptosis *via* two distinct events. The stimulated c-Jun N-terminal kinase (JNK) translocation to the nucleus elevates the pro-apoptotic gene expressions *via* triggering the c-Jun–dependent events. In contrast, the stimulated c-Jun N-terminal kinase (JNK) translocates to mitochondria and phosphorylates pro-apoptotic proteins, in that way antagonizing anti-apoptotic proteins and lastly exhibiting anti-apoptotic activity (5). Extracellular Signal-regulated Kinase (ERK) is a well-known anti-apoptotic protein that is regularly deregulated in cancer cells because of the mutations in several proteins. It primarily possesses an anti-apoptotic upshot *via* enhancing the anti-apoptotic protein expressions and hindering pro-apoptotic protein expressions (6).

Because of the vast developments in the medical field, the death rate of communicable diseases is decreased remarkably; meanwhile, the cancer-associated deaths were increased by 40% in recent decades (7). This is because of the fact that every tumor has its own characteristics, for example, tumor cells behave differently by means of multiplication, survival, and metastasis. In addition, tumor cells can acquire resistance to presently employed chemotherapeutic drugs (8).

The first-line treatment options for laryngeal cancer are chemo- and radiotherapy subsequent to the surgical removal. At present, the total laryngotomy is acknowledged as a most hopeful technique to treat laryngeal cancer, although it possesses some serious adverse effects, like problems with voice and swallowing (9). It was already proved that the promotion of tumor cell apoptosis is one of the hopeful techniques for cancer treatment (10). Although early-stage laryngeal cancer can be treated by radiotherapy or surgery, for the most of victims in the developed stage, there is still a lack of development standard care (11). Nevertheless, because of the lack of effectiveness of chemotherapy, patients have a poor prognosis due to the metastasis and local recurrence (12, 13). Consequently, the exploration of novel bioactive agents with the capacity of destroying the growth and metastasis of cancer cells is highly needed.

The medicinal plants have been enriched with immense bioactive compounds with anticancer properties (14–16). In addition, natural products are inexpensive when related to synthetic agents. Ononin is a natural isoflavone that is extensively dispersed in several food plants like *Astragalus membranaceus*, kudzu, broccoli, soybean, and lupine (17). It has already been described that ononin owns anti-inflammatory (18), antidiabetic (19), and antitumor activities (20). Meng et al. (21) mentioned that ononin treatment showed effective *in vitro* antiarthritic activity. Pan et al. (22) reported the cardioprotective properties of ononin. Ononin demonstrated an effective anti-angiogenic activity (23). Nonetheless, no reports were found on the antitumor property of ononin against laryngeal cancer. As a result, this research work was focused to explore the *in vitro* antitumor property of ononin against the laryngeal cancer Hep-2 cells through the ERK/JNK/p38 signaling inhibition.

## Materials and Methods

### Chemicals

Ononin (≥99.0%), Fetal Bovine Serum (FBS), Dimethyl Sulfoxide (DMSO), and other chemicals were purchased from Sigma-Aldrich (USA). The marker-specific kits for the biochemical examinations were attained from MyBioSource and Thermofisher (USA), respectively.

### Collection and Maintenance of Hep-2 Cells

Laryngeal cancer Hep-2 cells and normal HuLa-PC laryngeal cells were purchased from the American Type Culture Collection (ATCC) (USA). The collected cells were grown on a Dulbecco's Modified Eagle Medium (DMEM) enriched with Fetal Bovine Serum (FBS) (10%) at 37°C in a moistened CO_2_ (5%) incubator. The cultured cells were trypsinized after gaining the 80% confluency and utilized for further studies.

### Cytotoxicity Assay

The cytotoxic property of ononin against the Hep-2 and HuLa-PC cells was studied by 3-(4,5-dimethylthiazol-2-yl)-2,5-diphenyl tetrazolium bromide (MTT) cytotoxicity assay. Both cells were loaded separately on a 96-well plate at 5 × 10^3^ cells per well for 24 h at 37°C. Afterward, the medium containing cells was supplemented with the various concentrations (10–100 μM) of ononin for 24 h. Then, 20 μl of MTT along with Dulbecco's Modified Eagle Medium (DMEM) (100 µl) was mixed in all wells and stood for 4 h. The developed formazan crystals were liquefied using Dimethyl Sulfoxide (DMSO) (100 μl) and measured at 570 nm.

### Dual Acridine Orange/Ethidium Bromide (AO/EB) Staining

The apoptosis-inducing capacity of ononin on the Hep-2 cells was studied using AO/EB staining technique. Hep-2 cells were loaded onto the 24-well plate at 5 × 10^5^ cells per well. Then, Hep-2 cells were supplemented with the varied dosages (25 and 50 μM) of ononin for 24 h. Afterward, cells were stained by the addition of AO/EB (1:1) dye mixture (100 μg/ml) for 5 min, and lastly, cells were studied using a fluorescent microscope.

### Measurement of Reactive Oxygen Species (ROS)

The 2'-7'dichlorofluorescin diacetate (DCFH-DA) staining was employed to detect the ROS accumulation in control and ononin-supplemented Hep-2 cells. For this, Hep-2 cells were placed on a 24-well plate and treated with the ononin (25 and 50 μM) for 24 h. Cells were then stained by the addition of 10 μl of DCFH-DA stain for 1 h. Last, the production of ROS in the ononin-supplemented Hep-2 cells was examined under a fluorescent microscope.

### Mitochondrial Membrane Potential

The mitochondrial membrane potential (MMP) level in the ononin-supplemented and control Hep-2 cells was studied using Rh-123 staining. Hep-2 cells were placed on the 24-well plate and then treated with ononin (25 and 50 μM) and maintained for another 24 h at 37°C. Then, Rh-123 dye (10 μg/ml) was used to stain the cells for 30 min, and then, MMP was examined under a fluorescence microscope.

### Propidium Iodide Staining

The apoptotic levels were investigated using the propidium iodide (PI) staining technique. Hep-2 cells were loaded on a 24-wellplate for 24 h. Later, Hep-2 cells were supplemented with varied dosages (25 and 50 μM) of ononin for 24 h. Afterward, cells were stained with 5 µl of PI dye for 20 min, and then, apoptosis in the control and ononin-administered Hep-2 cells was monitored under a fluorescent microscope.

### Cell Adhesion Assay

The cell adhesion level in control and ononin-supplemented Hep-2 cells was studied, and, for this, Hep-2 cells were placed on a gelatin-coated plate and then supplemented with the various dosages (25 and 50 μM) of ononin for 60 min at 37°C. After that, cells were rinsed with saline and then trypan blue was utilized to stain the cells for the identification of adhesive levels and observed under an optical microscope.

### Measurement of Oxidative Stress and Antioxidants

The level of TBARS, glutathione (GSH), and SOD activity in the control and ononin (25 and 50 μM)–supplemented Hep-2 cells were assessed by assay kits using protocols described by the manufacturer (MyBioSource, USA).

### RT-PCR Analysis

The total RNA was separated from the Hep-2 cells using a TRIzol kit (Thermofisher, USA). After that, the isolated RNA was utilized to construct the cDNA using a PCR kit. The gene expressions were scrutinized by RT-PCR assay using manufacturer protocols (Takara, Japan). The primer sets are as follows: ERK1/2 sense: 5′-TCAAGCCTTCCAACCTC-3′, antisense: 5′-GCAGCCCACAGACCAAA-3′; JNK1/2 sense: 5′-GCCATTCTGGTAGAGGAAGTTTCTC-3′, antisense: 5′-CGCCAGTCCAAAATCAAGAATC-3′; p38 sense: 5′-AGGGCGATGTGACGTTT-3′, antisense: 5′-CTGGCAGGGTGAAGTTGG-3′; PI3K1/2 sense: 5′-GGACAATCGCCAATTCAG-3′, antisense: 5’-TGGTGGTGCTTTGATCTG-3’; and Akt sense: 5′-ATGAGCGACGTGGCTATTGTGAAT-3′, antisense: 5′-GAGGCCGTCAGCCACAGTCTGGATG-3′. The Glyceraldehyde-3-phosphate dehydrogenase (GADPH) was utilized as an internal control.

### Statistical Analysis

Data are analyzed using SPSS software. Values are deliberated as mean ± SD of triplicate results. Outcomes were scrutinized by one-way ANOVA and Tukey *post hoc* assay and p < 0.05 were fixed as significant.

## Results

### Ononin Treatment Decreased the Hep-2 Cell Viability and Did Not Reduce the Normal HuLa-PC Cell Viability

**Figure 1:**
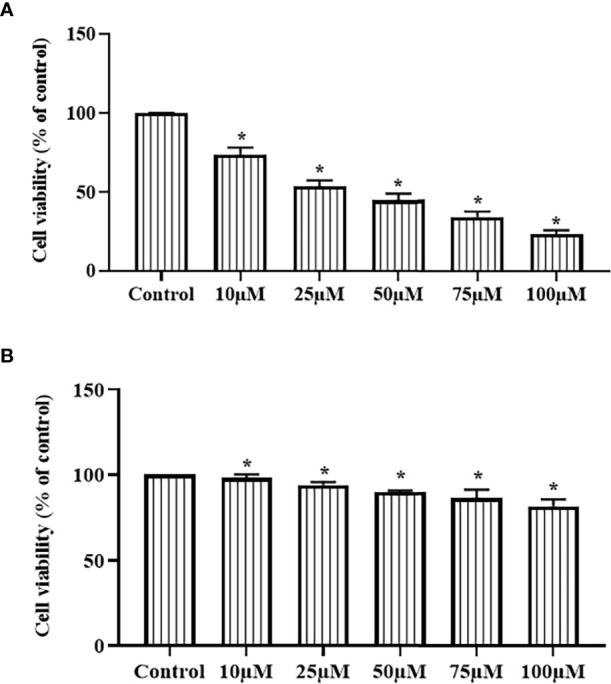
Effect of ononin on the cell viability of laryngeal cancer Hep-2 and normal HuLa-PC cells. The ononin treatment substantially decreased the viability of the laryngeal cancer Hep-2 cells **(A)** and did not affect that normal HuLa-PC cells **(B)**. The IC50 dose of ononin against Hep-2 cells was recorded at 25 µM. Outcomes were signified as mean ± SD of triplicate values. Outcomes were examined using one-way ANOVA and Tukey *post hoc* tests. “*” denotes p < 0.05 compared with control.

The effects of ononin treatment on the viabilities of Hep-2 and HuLa-PC cells were studied using an MTT assay, and the outcomes were given in **Figures 1A, B**. The ononin treatment at different dosages (10, 25, 50, 75, and 100 µM) appreciably diminished the Hep-2 cell viability (**Figure 1A**). However, the same concentrations of the ononin did not affect the viability of normal HuLa-PC cells (**Figure 1B**). A remarkable decrement was found in the viability of Hep-2 cells when treated with increased dosages of ononin. The Half-maximal inhibitory concentration (IC50) dose of ononin against the Hep-2 cells was found at 25 µM (**Figure 1A**). Consequently, 25 µM as an IC50 and 50 µM as a high concentration of ononin were selected for the further fluorescent staining assays.

### Ononin Treatment Increased the Apoptosis in the Hep-2 Cells

**Figure 2:**
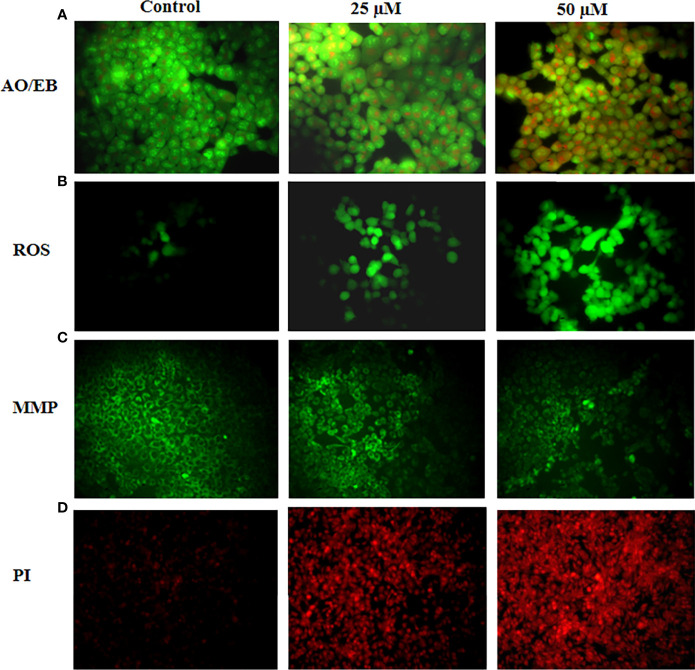
Effect of ononin on the apoptosis, ROS accumulation, and MMP level in the Hep-2 cells. The ononin (25 and 50 μM)–treated Hep-2 cells demonstrated the higher yellow and orange fluorescence than the control cells, which evidenced the occurrence of increased early and late apoptotic events **(A)**. The ononin (25 and 50 μM)–treated Hep-2 cells exhibit a bright green fluorescence, which confirms the improved ROS accumulation **(B)**. The Hep-2 cells treated with ononin (25 and 50 μM) revealed a depleted green fluorescence than the control cells, which evidenced the suppressed MMP level **(C)**. The increased red fluorescence was noted on the ononin (25 and 50 μM)–treated Hep-2 cells than the control cells, which proved the increased apoptotic cell death in the Hep-2 cells **(D)**.

The influence of ononin on the apoptosis in the Hep-2 cells was inspected by dual staining and findings were presented in **Figure 2A**. The ononin treatment (25 and 50 μM) remarkably augmented the apoptosis in the Hep-2 cells (higher yellow/orange fluorescence). The Hep-2 cells administered with ononin (25 and 50 μM) displayed an improved yellow/orange fluorescence than the control, which indicates the higher numbers of early and late apoptotic cells (**Figure 2A**).

### Ononin Treatment Elevated the ROS Generation in the Hep-2 Cells

**Figure 2B** reveals the outcomes of ononin on the ROS accumulation in the Hep-2 cells. The ononin treatment (25 and 50 μM) demonstrated the increased ROS generation in the Hep-2 cells. The Hep-2 cells treated with ononin (25 and 50 μM) revealed the intense green fluorescence that represents the occurrence of higher ROS accumulation (**Figure 2B**). When compared with the 25 µM treatment, the 50 µM ononin treatment drastically improved the ROS accretion in the Hep-2 cells.

### Ononin Treatment Reduced the MMP Level in the Hep-2 Cells

The effects of ononin administration on the MMP status of Hep-2 cells were scrutinized by Rh-123 staining, and findings were presented in **Figure 2C**. The ononin treatment (25 and 50 μM) appreciably decreased the MMP level in the Hep-2 cells when related to control cells. The Hep-2 cells administered with ononin (25 and 50 μM) displayed the reduced green fluorescence, which proves the depleted MMP (**Figure 2C**).

### Ononin Treatment Increased the Apoptosis in the Hep-2 Cells

The apoptotic inducing ability of ononin on the Hep-2 cells was studied using PI staining, and findings were portrayed in **Figure 2D**. As **Figure 2D** reveals, the ononin treatment (25 and 50 μM) remarkably improved the apoptosis in Hep-2 cells, which is confirmed by the improved red fluorescence. The Hep-2 cells administered with ononin (25 and 50 μM) displayed the intense red fluorescence that indicates the increased apoptosis (**Figure 2D**).

### Ononin Reduced the Cell Adhesion in the Hep-2 Cells

**Figure 3:**
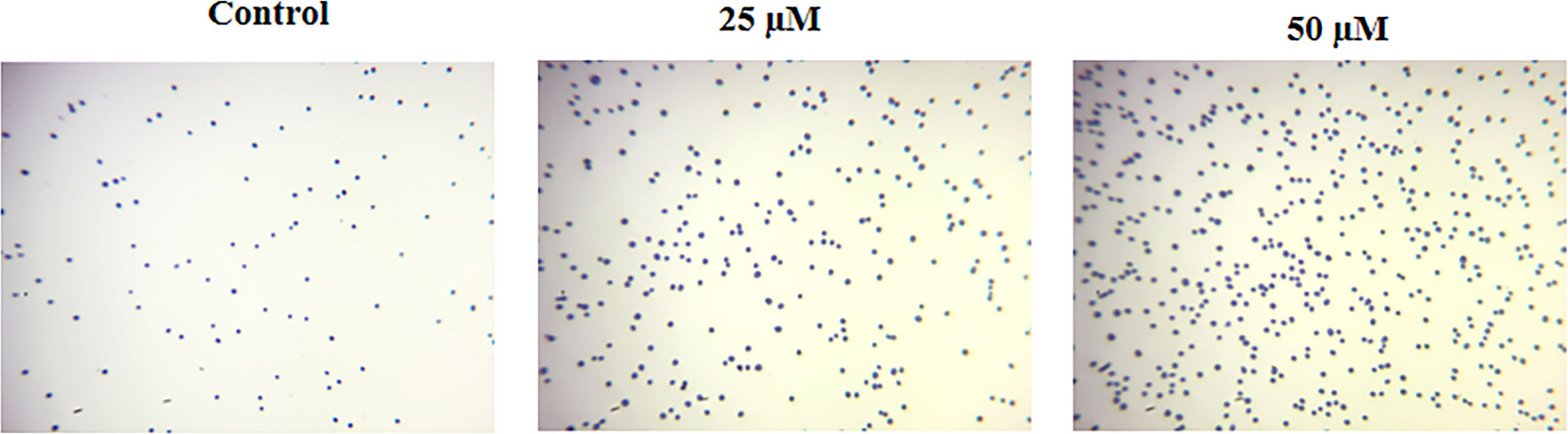
Effect of ononin on the cell adhesion of Hep-2 cells. The increased Trypan blue–stained cells in the ononin (25 and 50 μM)–supplemented Hep-2 cells displayed higher cell death when compared with control.

The impact of ononin on the cell adhesion of Hep-2 cells was inspected, and outcomes were represented in **Figure 3**. The ononin treatment effectively increased the cell death in the Hep-2 cells, which was witnessed by the Trypan blue staining. The cells administered with the ononin (25 and 50 μM) demonstrated the higher cell death when compared with control.

### Ononin Treatment Increased the TBARS and Depleted the Antioxidants in the Hep-2 Cells

**Figure 4:**
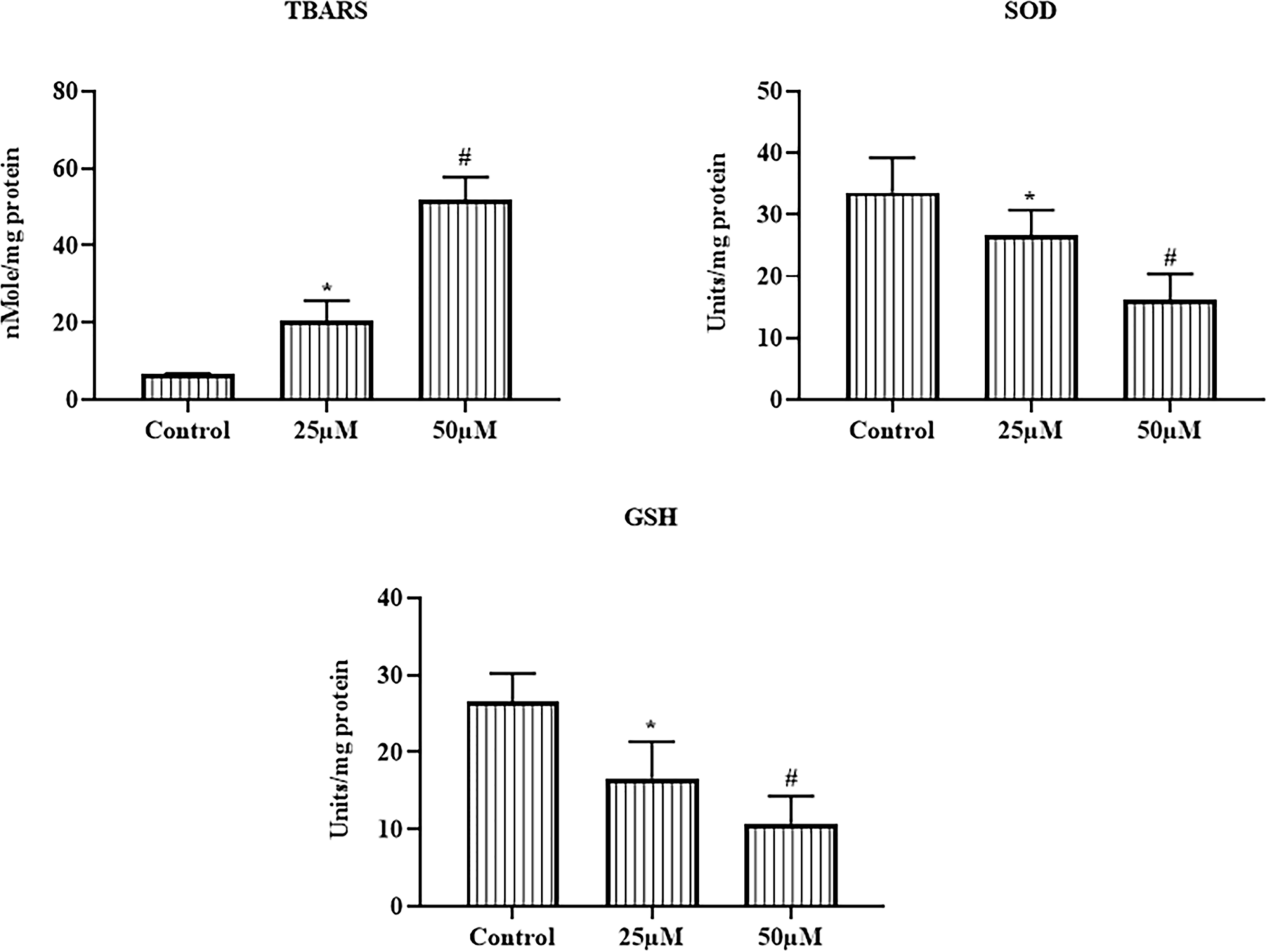
Effect of ononin on the TBARS and antioxidants in the Hep-2 cells. The ononin (25 and 50 μM)–administered Hep-2 cells demonstrated the improved TBARS and depleted GSH level and SOD activity than the control. Outcomes were signified as mean ± SD of triplicate values. Data were examined using one-way ANOVA and Tukey *post hoc* tests. “*” and “#” denote p < 0.05 compared with control.

The effects of ononin on the TBARS level, GSH level, and SOD activity were scrutinized using kits, and the results were displayed in **Figure 4**. The TBARS level was drastically elevated on the ononin (25 and 50 μM)–supplemented Hep-2 cells when related to the control. The ononin supplementation (25 and 50 μM) also depleted the GSH level and SOD activity in the Hep-2 cells (**Figure 4**). These findings evidenced that the ononin improved oxidative stress in the Hep-2 cells *via*, depleting the antioxidant mechanisms.

### Ononin Treatment Decreased the JNK/ERK/p38 Signaling Pathway in the Hep-2 Cells

**Figure 5:**
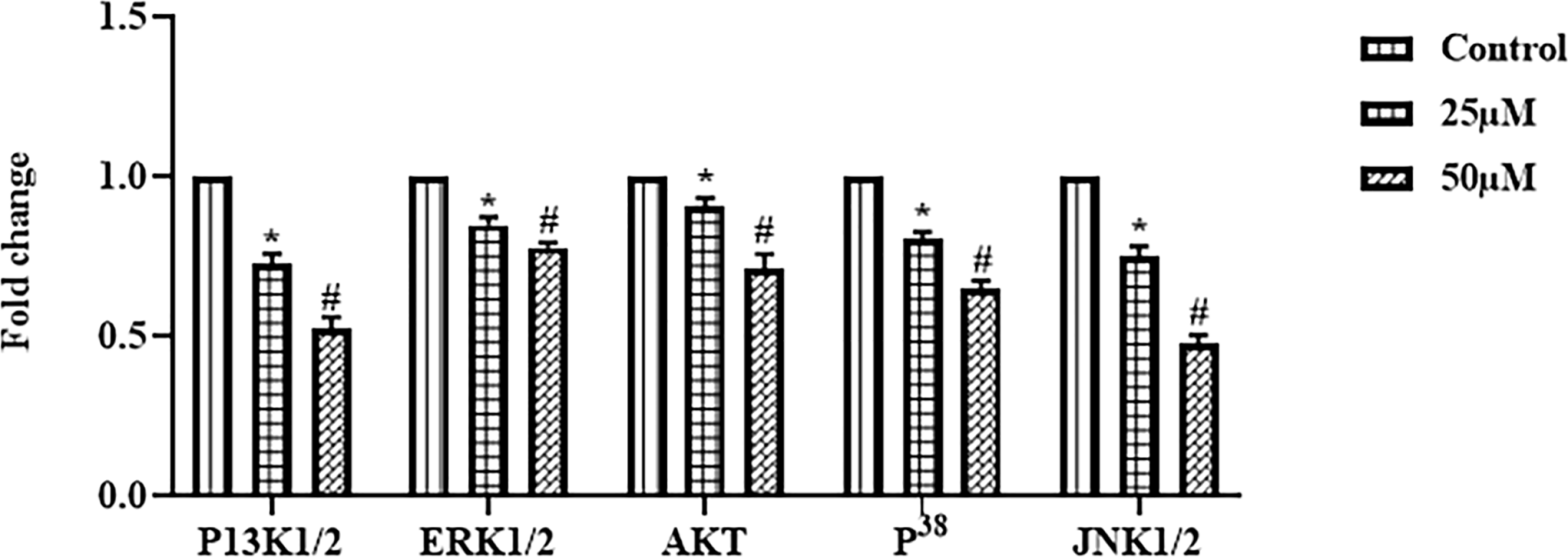
Effect of ononin on the JNK/ERK/p38 signaling pathway in the Hep-2 cells. The mRNA expressions of JNK1/2, ERK1/2, p38, and PI3K1/2 in the ononin (25 and 50 μM)–treated Hep-2 cells were remarkably decreased when compared with control. Outcomes were signified as mean ± SD of triplicate values. Data were examined using one-way ANOVA and Tukey *post hoc* tests. “*” and “#” denote p < 0.05 compared with control.

The expressions of JNK1/2, ERK1/2, p38, PI3K1/2, and Akt in the Hep-2 cells were inspected using RT-PCR, and outcomes were revealed in **Figure 5**. The ononin remarkably blocked the JNK/ERK/p38 signaling in the Hep-2 cells. The ononin treatment (25 and 50 μM) demonstrated the decreased expressions of JNK1/2, ERK1/2, p38, and PI3K1/2 in the Hep-2 cells. The ononin also improved the Akt expression in the Hep-2 cells (**Figure 5**). These findings demonstrated that the ononin inhibited the JNK/ERK/p38 signaling pathway in the Hep-2 cells.

## Discussion

Laryngeal cancer is a general head and neck malignant tumor with a poor prognosis and survival rate (24). Lifestyle habits comprising drinking and smoking, biliary tract ailments, and gastroesophageal reflux can elevate the laryngeal cancer risks (25, 26). Presently, surgical resection along with radio/chemotherapy is the first-line treatment approach for laryngeal cancer. The patients with developed stage are susceptible to recurrence and metastasis subsequently surgery, which results in a poor prognosis. In addition, the adverse effects of chemotherapeutic agents also restrict their anticancer efficiency (27). Hence, the current study focuses to explore the *in vitro* antitumor action of ononin against the laryngeal cancer Hep-2 cells. Our findings confirmed that the ononin substantially inhibited the Hep-2 cell growth. In addition, the ononin did not affect the viability of normal HuLa-PC cells, which proves the selective toxicity of ononin against laryngeal cancer cells.

Apoptosis is a highly regulated cell necrotic event that performs critical functions in manifold biological mechanisms in normal tissues. The faults in apoptosis could enhance the tumor progression and make tumor cells highly resistant to therapy. In this aspect, the elusion of apoptosis is a remarkable phenomenon in cancers (28). Essentially, cancer cells display lessened apoptotic events that lead to assisting the progression and metastasis of cancer cells (29, 30). Tumor cells evade the normal apoptosis and continue to multiply, obstructing the normal cells or tissue functions, which can lead to death. Thus, stimulating apoptosis has been regarded as a hopeful option to hinder tumors (31). Furthermore, the important aim of clinical oncology is the improvement of treatment strategies enhancing the potential removal of tumor cells *via* triggering apoptosis (32). In this research, we witnessed that the ononin triggered the apoptotic cell death in the Hep-2 cells, which is witnessed by the outcomes of dual staining and PI (**Figures 2A, D**).

Several investigations were proved that the array of anticancer agents triggers apoptosis *via* its oxidative effects, like depleting cellular antioxidant mechanisms and/or elevating ROS accumulation (33). Although overaccumulation of ROS is tightly connected to mitochondrial dysfunction, it participates in the extrinsic and intrinsic cascades. In addition, the accumulation of aberrant ROS has straight connections in causing the oxidative injury of DNA (34). Hence, these annotations propose that an elevation in ROS accumulation in tissues/cells is an imperative phenomenon for enhancing tumor cell necrosis. Regulating intracellular ROS status could proficiently kill tumor cells and suppress the adverse effects of radio/chemotherapy, and it is presently regarded as the primary means of tumor management (35). Several previous studies already demonstrated that many natural compounds are well known to increase the intracellular ROS production and lead to cell death in cancer cells (36–38). Likewise, we found that the ononin treatment substantially augmented the intracellular ROS status in the Hep-2 cells, thereby leading to oxidative stress mediated cell death (**Figure 2B**).

PI3K/AKT axis is participated in mediating the cell multiplication, cell cycle, and apoptosis (39). In cancer cells, the PI3K axis is highly stimulated (40). The stimulated AKT phosphorylate Bad enhances the antiapoptotic protein expressions, in that way hindering apoptosis *via* the mitochondrial pathway (41). In addition, AKT can hinder apoptosis *via* triggering various signaling proteins like Nuclear factor kappa B (NF-κB) (42). Consecutively, inhibiting this signaling cascade may efficiently enhance the tumor cell apoptosis to exhibit anticancer activity. It was already stated that stimulation of the PI3K/Akt cascade not only improves the multiplication and metastasis of tumor cells but also provokes the chemoresistance toward chemotherapy (43–45). Interestingly, our outcomes revealed that the PI3K/AKT axis in the Hep-2 cells was substantially blocked by the ononin (**Figure 5**).

The GSH and SOD are the prime antioxidants that guard the cells/tissues against oxidative stress (46). The basal status of ROS could sustain the normal cell homeostasis; low and chronic status of ROS enhances mitosis and improves genomic uncertainty to stimulate the incidence and development of cancers (47); high and acute ROS levels damage macromolecules and consequently provoke apoptosis, ferroptosis, and necrosis. Hence, the elevated ROS levels in cancer cells and defects in antioxidant systems make tumor cells highly vulnerable to ROS inflection (48). Our findings from the current study proved that the ononin treatment remarkably enhanced the TBARS level and depleted the GSH level and SOD in the Hep-2 cells, thereby facilitating oxidative stress-mediated cell death (**Figure 4**).

As described earlier, MAPK signaling cascades mediate several cellular events like apoptosis. Manifold reports have recognized that JNK/p38 MAPK signaling axis is actively participated in cell necrosis, whereas the Extracellular Signal-regulated Kinase (ERK) cascade is connected with cell survival (49). As described, oxidative stress triggers c-Jun N-terminal kinase (JNK) expression and deactivates the anti-apoptotic protein expressions, although it stimulates the pro-apoptotic protein expressions (50). The stimulation of c-Jun N-terminal kinase (JNK) and p38 is essential for apoptosis, and ERKs are connected to the tumor cell multiplication and resistance toward apoptosis. The p38 MAPK cascade is stimulated by inflammatory mediators, environmental stress, and several other mitogens (51). The c-Jun N-terminal kinase (JNK) signaling axis actively participates in manifold cellular events where it mediates a variety of cellular mechanisms like multiplication, apoptosis, differentiation, and others (52, 53). The Extracellular Signal-regulated Kinase (ERK) signaling cascade was tightly connected to the multiplication, variation, and apoptosis in tumor cells (54). The abnormal stimulation of the Extracellular Signal-regulated Kinase (ERK) cascade is essential for the incidence and development of several tumors. Hence, novel agents that target the Extracellular Signal-regulated Kinase (ERK) signaling axis can signify the efficient and notable active drugs for tumor treatment (55).

The improvement of tumor cell resistance demonstrates the main difficulty during the monotherapy with ERK/MAPK inhibitors. The improvement of resistance frequently arises due to the ERK/MAPK crosstalk with other signaling cascades like PI3K/Akt signaling. Furthermore, many reports highlight the critical functions of activating the ERK/MAPK cascade during cell necrosis initiation in a wide variety of tumor cells (56). Interestingly, we found that the mRNA expressions of JNK1/2, ERK1/2, and p38 in the Hep-2 cells decreased by the ononin administration (**Figure 5**). These findings suggest that the ononin can block the JNK/ERK/p38 signaling in the Hep-2 cells.

## Conclusion

Together, our results confirmed that ononin could prevent cell growth, stimulate cytotoxicity, and trigger apoptosis in the Hep-2 cells. Most prominently, our outcomes revealed that ononin can block the JNK/ERK/p38 signaling axis in the Hep-2 cells and induce apoptosis. However, the exact therapeutic roles of ononin against laryngeal cancer need to be confirmed in the future with further research. In the future, additional works in this context are needed for the development of a new chemotherapeutic agent for the management of laryngeal cancer.

## Data Availability Statement

The original contributions presented in the study are included in the article/supplementary material. Further inquiries can be directed to the corresponding author.

## Author Contributions

BY and JM performed experiments and analyzed results; ZL analyzed results; BY, YL, and XH prepared figures and first draft; BY, JM, ZL, YL, and XH edited manuscript. All authors contributed to the article and approved the submitted version.

## Conflict of Interest

The authors declare that the research was conducted in the absence of any commercial or financial relationships that could be construed as a potential conflict of interest.

## Publisher’s Note

All claims expressed in this article are solely those of the authors and do not necessarily represent those of their affiliated organizations, or those of the publisher, the editors and the reviewers. Any product that may be evaluated in this article, or claim that may be made by its manufacturer, is not guaranteed or endorsed by the publisher.
